# Factors affecting pharmacokinetic variability of oral topotecan: a population analysis

**DOI:** 10.1038/sj.bjc.6601469

**Published:** 2004-01-20

**Authors:** F Léger, W J Loos, J Fourcade, R Bugat, M Goffinet, R H J Mathijssen, J Verweij, A Sparreboom, E Chatelut

**Affiliations:** 1 EA3035, Institut Claudius-Regaud, 20-24 rue du Pont-St-Pierre, F-31052 Toulouse, France; 2 Department of Medical Oncology, Erasmus MC – Daniel den Hoed Cancer Center, Rotterdam, the Netherlands

**Keywords:** pharmacokinetics, drug monitoring, topoisomerase I inhibitor

## Abstract

The aim of this study was to characterise the pharmacokinetics of the anticancer agent topotecan, and explore the influence of patient covariates and interoccasion variability on drug disposition. Data were obtained from 190 patients who received the drug as a 30-min infusion (*N*=72) or orally (*N*=118). The population model was built with the use of NONMEM to identify candidate covariates, and obtain models for clearance (CL) and volume of distribution. The final models were based on first-order absorption with lag-time (oral data), and a two-compartment model with linear elimination from the central compartment. The Cockcroft–Gault creatinine clearance (CrCl) and WHO performance status (PS) were the only significant covariates: CL=(12.8+2.1 × CrCl) × (1−0.12 × PS). For the volume of distribution, a correlation was found between body weight and the central volume (V1)=0.58 × body weight. Based on the structural models, a limited-sampling strategy was developed with minor bias and good precision that can be applied *a posteriori* using timed samples obtained at 1.5, and 6 h after the administration of topotecan. In conclusion, a population pharmacokinetic model for topotecan has been developed that incorporates measures of renal function and PS to predict CL. In combination with drug monitoring, the limited sampling strategy allows individualised treatment for patients receiving oral topotecan.

Topotecan (9-dimethylaminomethyl-10-hydroxycamptothecine) is a water-soluble semisynthetic analogue of camptothecin that binds to topoisomerase I–DNA complexes, leading to single-stranded, protein-associated DNA breakage and cellular cytotoxicity. The drug is poorly bound to plasma proteins, but is present under open hydroxy acid and closed lactone forms within the plasma, according to a relatively constant ratio determined primarily by pH (Herben *et al*, 1996). Topotecan (Hycamtin®) has demonstrated antitumour activity in several tumour types, including ovarian cancer and small-cell lung cancer (Brogden and Wiseman, 1998; Ormrod and Spencer, 1999). The drug presents the requirements for considering therapeutic drug monitoring; its interindividual pharmacokinetic variability is significant and relationships are described between the area under the plasma concentration–time curve (AUC) and its dose-limiting toxicity (i.e. neutropenia) (Grochow *et al*, 1992; Rowinsky *et al*, 1994; van Warmerdam *et al*, 1995).

Previous investigations have shown that, by considering patient covariates (i.e. body weight, serum creatinine, and sex), the accuracy of prediction of topotecan clearance (CL) can be improved only partly (Gallo *et al*, 2000; Montazeri *et al*, 2000; Mould *et al*, 2002). Moreover, clinical protocols are based on repeated daily administrations (usually, 5 consecutive days) giving the opportunity to perform a dose adjustment according to the observed exposure on Day 1. The intrapatient pharmacokinetic variability of topotecan following the intravenous (i.v.) or oral administration is limited (Loos *et al*, 2000; Montazeri *et al*, 2000). Pharmacokinetically guided dosing methods have been performed both in adults and in children for topotecan given by i.v. administration (Stewart *et al*, 1994; Montazeri *et al*, 2002). However, the oral route of administration for topotecan is attractive as it allows dosing for prolonged periods of time in an outpatient setting. The oral bioavailability of topotecan is approximately 30–40% (Schellens *et al*, 1996; Kruijtzer *et al*, 2002). These previous data were obtained using conventional pharmacokinetic analyses performed in only limited number of patients (not more than 12). In contrast, population pharmacokinetic analyses present the advantage of the ability to discriminate between the several sources of variability.

Here, a large data set of topotecan plasma concentrations was built from data obtained after various schedules of i.v. and oral administration, either given as a single agent or in combination with cisplatin. The goals of the current population pharmacokinetic analysis were (i) to quantify simultaneously the different sources of variability on the pharmacokinetic parameters; (ii) to update a previous covariate model for predicting topotecan pharmacokinetic parameters; and (iii) to develop a limited sampling strategy based on Bayesian analysis that can be applied to oral administration of topotecan.

## PATIENTS AND METHODS

### Patient population

Data were obtained from 190 patients who participated in five separate clinical trials (173 patients), or who had a drug monitoring for other reasons (17 patients). All patients provided written informed consent to participate in the respective protocols, as approved by the local or regional ethical committees. The patient characteristics are shown in Table 1

**Table 1:** Characteristics of the 190 patients studied

**Characteristics**	**Mean (range)**
Age (year)	55 (18–76)
BSA (m^2^)^a^	1.80 (1.36–2.44)
Body weight (kg)	70 (42–117)
Serum creatinine (*μ*mol l^−1^)	87 (41–162)
Creatinine clearance (ml min^−1^)^b^	80 (33–167)
	**Number**
WHO performance status: 0/1/2/3	79/98/11/2
Previous chemotherapy	141
*Cisplatin pretreatment*	
During previous regimen	115
On day 1 of topotecan treatment	30

a Calculated according to the Dubois formula.

b Calculated according to the Cockcroft–Gault equation.

, and a brief listing of the study designs is given in Table 2

**Table 2:** Characteristics of clinical studies used in pooled analysis

**Route**	***n***	**Indication and study type**	**Duration of therapy**	**Dose (mg m^−2^ per day)**
i.v.	39	Ovarian cancer; individual dosing^a^	Once daily for 5 days every 3 week	0.2–2.4
i.v.	16	Solid tumour; phase I	Once daily for 5, 7, 10, or 13 days	0.2–1.0
Oral	55	Solid tumour; phase I^b^	Once or twice daily for 5, 10, or 21 days	0.15–2.7
Oral	55	Solid tumour; phase I combined with cisplatin^c^	Once daily for 5 days every 3 week	0.75
Oral	8	Solid tumour; combined with cisplatin	Once daily for 5 days every 3 week	0.45–0.6
i.v.	17	Ovarian cancer; therapeutic drug monitoring	Once daily for 5 days every 3 week	0.2–1.5

a Montazeri *et al* (2002).

b Gerrits *et al* (1999).

c Loos *et al* (2000).

*n*=number of patients.

. Topotecan was administered i.v. (72 patients) over 30 min daily for 5–13 consecutive days at a dose ranging from 0.2 to 2.4 mg m^−2^ day^−1^, or was administered orally (118 patients) using drug formulated in gelatine capsules at dose levels ranging from 0.15 to 2.7 mg m^−2^ day^−1^ for 5–21 consecutive days.

### Blood sampling and plasma topotecan analysis

The total number of available plasma samples was 2064; two to 21 per patient (median, 15) in cycle 1 and two to 18 per patient (median, 7) in cycle 2. The pharmacokinetic analysis was performed on day 1 of cycle 1 (190 patients), after the last administration of cycle 1 (175 patients), on day 1 of cycle 2 (93 patients), and after the last administration of cycle 2 (47 patients).

Topotecan concentrations in the plasma were determined as the total of lactone and carboxylate forms (i.e. as total drug) using high-performance liquid chromatography (HPLC) as previously described (de Jonge *et al*, 2000; Montazeri *et al*, 2002). The lower limits of quantification were 0.5 (Toulouse) and 0.1 ng ml^−1^ (Rotterdam), and the mean percentage deviation from nominal values (accuracy) and precision (within-run and between-run variability) of quality control samples simultaneously run during the analysis was always less than 15%.

### Pharmacokinetic analyses

Concentration *vs* time profiles of total topotecan in plasma were analysed using NONMEM (Beal and Sheiner, 1982) (version V, level 1.1) with the first-order conditional estimation method and the PREDPP package running on a personal computer. A proportional error model was used for the interpatient variabilities.

### Relationships between covariates and pharmacokinetic parameters

In total, 17 patients’ covariates were tested: age, albuminemia, bilirubinaemia, body surface area (BSA), body weight, cisplatin pretreatment (either as previous regimen or concurrent regimen on day 1 and cisplatin), Cockcroft–Gault creatinine clearance (CrCl), gender, haemoglobinaemia, proteinaemia, serum alanine transaminase (ALT), serum aspartate transaminase (AST), serum creatinine, and WHO performance status (PS). Interoccasion variability (Karlsson and Sheiner, 1993) was used in order to take into account random variability on pharmacokinetic parameters between the first and the last day of topotecan administration. In analysing the data, NONMEM computed the value of a statistical function (i.e. the minimal value of the objective function), which is equal to minus twice the log likelihood. For testing of the covariates, the different models were compared using the approximation to the *χ*^2^ distribution of the objective function value of the reduced model minus that of the full model. The number of degrees of freedom is equal to the difference in the number of parameters between two nested models.

### Intraindividual variability between cycles 1 and 2

All data (i.e. from day 1 of cycle 1 to the last day of topotecan administration of cycle 2) were analysed to evaluate the intercycle variability. The final covariate model obtained during the first step of the analysis was used. Interoccasion variability was used in order to take into account random variability between cycles 1 and 2.

### Development of a limited sampling strategy for oral topotecan

Seven times intervals for the blood collection were composed from the oral data: (0.9–1.2) (referred to 1 h henceforth), (1.4–1.6) (1.5 h), (1.8–2.2) (2 h), (2.8–3.3) (3 h), (3.9–4.1) (4 h), (5.9–6.3) (6 h), and (7.8–8.2) (8 h) h after oral administration. Among the data from the 118 patients who received topotecan orally, two groups of patients (*N*=15, each) were randomly constituted: a training group and a validation group. Bayesian estimation was performed to determine the topotecan AUC from a limited number of samples. The data (day 1 of cycle 1) from the training group were used to select the best three-sample schedule among 23 different combinations of three samples. The predictive performance of this schedule was prospectively evaluated using the data of the validation group. For a patient j of the test group, the relative prediction error, pej (%), for AUC was defined as follows: pej (%)=(AUC_LSS_−AUC) × 100/AUC, where AUC_LSS_ is the Bayesian estimate of AUC for patient j, AUC is the actual AUC that was obtained using all data points. The predictive performance of the formula was evaluated by computing the mean relative prediction error (me%=*N*^−1^∑_*j*=1^*N*^_ (pe_j_) where *N* is the number of patients=15) as a measure of bias and the root mean squared relative prediction error (rmse%=[*N*^−1^∑_*j*=1^*N*^_ (pe_j_^2^)]^1/2^) as an assessment of precision.

## RESULTS

**Figure 1:**
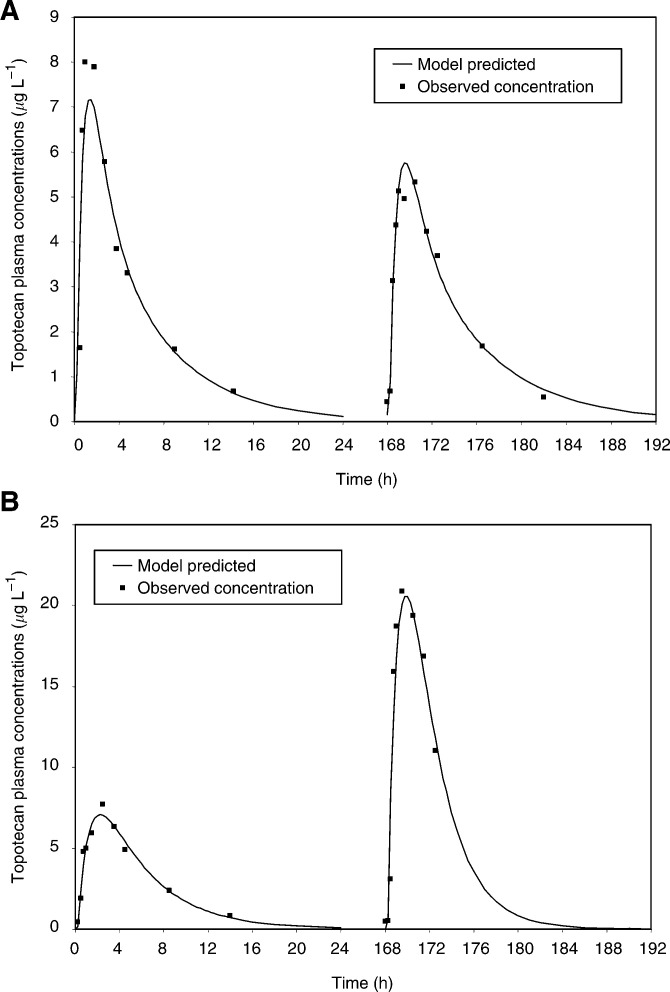
Observed topotecan concentrations (data points) and model-predicted concentrations using the interoccasion variability option: data from one patient with minimal (−9%, **A**) or large (+104%, **B**) change of AUC after administration of the same dose at days 1 and 5 (1.5 mg m^−2^).

The development of the structural pharmacokinetic model indicated that a first-order absorption with lag-time (oral data), and a two-compartment model with linear elimination from the central compartment best fit the topotecan plasma concentrations *vs* time profiles. A combination model (i.e. additive plus proportional) was used for the residual variability with specific values for oral and i.v. data. Figure 1

shows two representative examples of the fit of the topotecan plasma concentrations observed after oral administration: one with limited interday variability, the second with large interday variability. In terms of interindividual variability, by considering the data at cycle 1, the AUC, normalised to dose, presented a 4.8-fold and a 7.6-fold variability for i.v. and oral data, respectively. In terms of interday variability (also corresponding to the intrapatient variability within cycle 1), the percentage of change in AUC, normalised to dose and expressed as the root mean squared relative prediction error, was 22.5 and 43.0% for i.v. and oral data, respectively.

### Relationships between covariates and pharmacokinetic parameters

During individual testing of the 17 covariates, two covariates (i.e. the Cockcroft–Gault CrCl, and the WHO PS) were significantly correlated with topotecan CL. For the volumes of distribution, a correlation was found between body weight and the central volume (V1) (a correlation between BSA and V1 was significant, but weaker). No liver function test (i.e. serum bilirubin, ALT, AST) was significantly correlated with bioavailability (F). Testing of the intermediate model led to the final model that is presented in Table 3. The proportional part and the additive part corresponding to the residual variability associated with the final covariate model were 11 and 0.64 *μ*g l^−1^ for i.v. data, and 17% and 0.09 *μ*g l^−1^ for oral data, respectively. Difference corresponding to the additive part between i.v. and oral data may be, at least partly, due to the difference of limit of HPLC quantification between Toulouse and Rotterdam analyses. Indeed, i.v. and oral data matched with Toulouse and Rotterdam HPLC measurements, respectively.

### Intraindividual variability between cycles 1 and 2

The covariate model was used with updated values for CrCl, body weight, and PS at cycle 2. The coefficients of variation corresponding to the intercycle variabilities on topotecan CL, central volume of distribution (V1), and bioavailability (F) were 18, 31, and 22%, respectively. The final covariate models obtained from the data from both cycles (i.e. CL=(11.4+2.5 × CrCl) × (1−0.06 × PS) and V1=0.50 × body weight) were similar to those from the data of cycle 1.

### Limited sampling strategy to estimate topotecan exposure after oral administration

The structural model was that corresponding to the final covariate model except that PS was not taken into account. By analysis of the data of the training group, bias and precision corresponding to the 23 three-sample schedules ranged between −4 and +11, and 8 and 21, respectively. The best schedule considering both criteria was 1.5, 4, and 6 h, with a bias and precision of +0.6 and 8.6%, respectively. The three combinations of two-sample schedules corresponding to these three time intervals were then tested, and the best prediction corresponded to the two-sample schedule using the data obtained at 1.5 and 6 h after oral administration. These two schedules (i.e. 1.5–4–6 and 1.5–6) were evaluated prospectively using the data of the validation group. Predictive performances are shown in Table 4

**Table 3:** Mean, interindividual and interday variability, and covariate models of the topotecan pharmacokinetics parameters at cycle 1

	**Mean (±95% CI^b^)**	**ΔOBJ^d^**	***P***	**%CV^c^**
*Final covariate model*^a^				
CL(l h^−1^)=(*θ*1+*θ*2 × CrCl) × (1−*θ*3 × PS)	*θ*1=12.8 (4.8); *θ*2=2.1 (1.0); *θ*3=0.12 (0.09)			29
Interday variability of CL				18
				
Central volume: V1 (l)=*θ*4 × body weight	*θ*4=0.58 (0.13)			39
Interday variability of V1				49
				
Peripheral volume: V2 (l)=*θ*5	*θ*5=45.5 (7.0)			46
Intercompartmental clearance: *Q*(l h^−1^)=*θ*6	*θ*6=49.2 (16.9)			80
Bioavailability: F(%)=*θ*7	*θ*7=32.4 (3.9)			22
Interday variability of F				28
				
Constant rate of absorption: Ka(h^−1^)=*θ*9	*θ*9=1.7 (0.6)			64
Lag time (h)=*θ*10	*θ*10=0.17 (0.03)			13
				
				**%CV^b^**
*Alternative covariate models*				
CL=*θ*1	*θ*1=20.1 (2.3)	+62	<0.001	39
CL=*θ*1+*θ*2 × CrCl	*θ*1=11.8 (4.5); *θ*2=1.90 (1.16)	+20	<0.001	20
CL=*θ*1 × (1−*θ*3 × PS)	*θ*1=21.8 (3.1); *θ*2=0.11 (0.10)	+47	<0.001	37
V1=*θ*4	*θ*4=35.2 (10.1)	+27	<0.001	33

a CrCl for Cockcroft–Gault creatinine clearance (L h^−1^), PS for WHO performance status.

b Confidence interval.

c Coefficient of variation for interindividual variability (not explained by the covariate, if any) or interday variability.

d Change in objective function by comparison with the final covariate model.

. Figure 2

**Figure 2:**
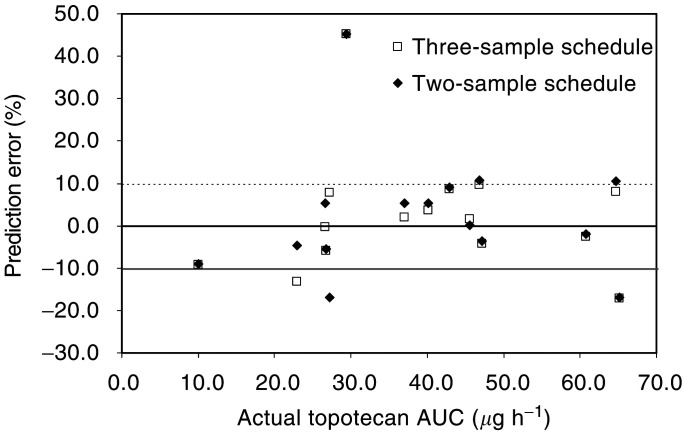
Ratio plot for prospective evaluation of the limited sampling applied to the oral administration of topotecan. Plasma AUC were estimated on concentrations at times 1.5 and 6 h (two-sample schedule), 1.5, 4, and 6 h (three-sample schedule) after oral administration using NONMEM.

compares AUC determined by considering all data concentrations and that obtained from either the three- or the two-sample schedule.

## DISCUSSION

Oral chemotherapy represents a fundamental change in contemporary oncology practice, driven by patient convenience (Liu *et al*, 1997), pharmacoeconomic issues, and the potential for improved patient quality of life (DeMario and Ratain, 1998). The oral route for administration is particularly attractive in the case of topotecan. Oral topotecan has demonstrated activity and tolerability similar to i.v. topotecan in chemosensitive small-cell lung cancer (von Pawel *et al*, 2001) and relapsed epithelial ovarian cancer (Gore *et al*, 2002), and thus seems to offer patients a convenient alternative to i.v. therapy. Each topotecan treatment cycle is composed of 5 days of administration and even more prolonged schedule has been considered (Hochster *et al*, 1999).

One potential problem in substituting i.v. by oral administration is a greater degree of variability in AUC because of inter- and intraindividual differences in drug absorption. While this is observed for all classes of oral drugs, the issue is especially critical for cancer chemotherapy, in which a narrow therapeutic index is frequently observed. In the current study, we applied the population approach for quantifying the several sources of pharmacokinetic variability using the NONMEM on a large data set. Then the mean observed bioavailability of oral topotecan was found to be 32.4%, which retrospectively explained the better haematological tolerance of 2.3 mg m^−2^ day^−1^ given orally than that of 1.5 mg m^−2^ day^−1^ i.v. (von Pawel *et al*, 2001; Gore *et al*, 2002). The interindividual variability in the bioavailability was estimated to be 22%, whereas the intrapatient variability of F was also limited (28% for the interday, and 22% for the intercycle variability). Regardless, these variations represent a further factor of variability in topotecan AUC when compared with the i.v. route of administration. The limited bioavailability is likely due to the affinity of topotecan for drug transporter (such as breast cancer resistance protein) at the small intestine level (Kruijtzer *et al*, 2002), and not to significant first-pass hepatic metabolism. Furthermore, we did not observe any correlation between F and liver function tests (serum bilirubin, ALT, AST).

Several studies have shown the correlation between topotecan plasma exposure and the percentage of decrease in white blood cells, and that severe haematological toxicity is often associated with the recommended dose of topotecan. Hence, determination of factors that can explain interindividual pharmacokinetic variability may be useful for individualised dosing strategies for this drug. The covariate analysis performed here represents a refinement of our previous study (Montazeri *et al*, 2000). The final model (Table 3

**Table 4:** Predictive performance of Bayesian estimation of AUC of topotecan given orally with limited sampling schedules tested in 15 patients (*me* mean relative prediction error, *rmse* root mean squared relative prediction error)

**Sampling time intervals (hours postadministration)**	**Bias, me (%)**	**Precision, rmse (%)**	**Range of prediction error**
1.5, 4, and 6 h	+2.3	9.3	−17.0–+45.2
1.5, and 6 h	+2.2	10.0	−17.0–+45.3

) is consistent with those proposed by Gallo *et al* (2000), and Mould *et al* (2002), who also analysed data collected in different phase I trials. The data may be considered as physiological, with 12.8 l h^−1^ corresponding to the non-renal CL, and the coefficient 2.1 for CrCl illustrates that renal elimination of topotecan exceeds the glomerular filtration rate due to tubular secretion of the drug (Zamboni *et al*, 1998). We also observed a limited but significant relationship between topotecan and WHO PS; a patient with a PS of 2 has a CL, which is decreased on average by 24% compared to a patient with a PS of 0. Mould *et al* (2002) observed a similar impact with ECOG PS. With respect to these consistent results, topotecan dosing should be individualised according to these two covariates (i.e. CrCl and PS) rather than the currently used approach based on BSA alone. The covariate PS was previously tested during the two other previous studies, but was not found to be as significant; it is likely due to their smaller numbers of patients (i.e. 82, Gallo *et al*, 2000 and 31, Montazeri *et al*, 2000). Lastly, it is interesting to note that topotecan CL was not modified by cisplatin treatment at day 1 of topotecan cycle, confirming the previous analysis performed at the Rotterdam Cancer Institute (de Jonge *et al*, 2000).

A method to control the plasma exposure of drugs given by multiple consecutive days is to perform a drug monitoring and then to adjust the dose according to the target AUC values. For oral topotecan, this method would allow to annul the impact of interindividual variability on CL and bioavailability. The limited number of blood samples would allow generalisation of this drug monitoring. The three-sample schedule and Bayesian method of analysis gave precise and unbiased estimates of the topotecan AUC after oral administration. The two-sample schedule may also be used with comparable performance. Only one discordant value was obtained by both schedules. However, the intrapatient variability (present mainly for bioavailability as shown by interday variability of 28% for F) will limit the possibility to extrapolate overall AUC from a unique day of drug monitoring. Again, the limited number of samples per day may help to reiterate this exploration.

In conclusion, this analysis has quantified the several sources of variability in topotecan AUC, depending on the route of administration and patient covariates. A limited sampling strategy would allow performing drug monitoring and individual dose adjustment for oral topotecan.
